# Spatial Calibration of Humanoid Robot Flexible Tactile Skin for Human–Robot Interaction

**DOI:** 10.3390/s23094569

**Published:** 2023-05-08

**Authors:** Sélim Chefchaouni Moussaoui, Rafael Cisneros-Limón, Hiroshi Kaminaga, Mehdi Benallegue, Taiki Nobeshima, Shusuke Kanazawa, Fumio Kanehiro

**Affiliations:** 1 National Institute of Advanced Industrial Science and Technology (AIST), Tsukuba 305-8560, Japan; selim.cm@mailo.com (S.C.M.); hiroshi.kaminaga@aist.go.jp (H.K.); mehdi.benallegue@aist.go.jp (M.B.); nobeshima.taiki@aist.go.jp (T.N.); kanazawa-s@aist.go.jp (S.K.); f-kanehiro@aist.go.jp (F.K.); 2 CNRS-AIST Joint Robotics Laboratory (JRL), AIST, Tsukuba 305-8560, Japan; 3 Human Augmentation Research Center (HARC), AIST, Kashiwa 277-0882, Japan

**Keywords:** tactile sensing, flexible sensors, sensor calibration, surface fitting, physical human–robot interaction, sensor integration

## Abstract

Recent developments in robotics have enabled humanoid robots to be used in tasks where they have to physically interact with humans, including robot-supported caregiving. This interaction—referred to as physical human–robot interaction (pHRI)—requires physical contact between the robot and the human body; one way to improve this is to use efficient sensing methods for the physical contact. In this paper, we use a flexible tactile sensing array and integrate it as a tactile skin for the humanoid robot HRP-4C. As the sensor can take any shape due to its flexible property, a particular focus is given on its spatial calibration, i.e., the determination of the locations of the sensor cells and their normals when attached to the robot. For this purpose, a novel method of spatial calibration using B-spline surfaces has been developed. We demonstrate with two methods that this calibration method gives a good approximation of the sensor position and show that our flexible tactile sensor can be fully integrated on a robot and used as input for robot control tasks. These contributions are a first step toward the use of flexible tactile sensors in pHRI applications.

## 1. Introduction

The interaction between robots and humans—often referred to as human–robot interaction (HRI)–relies on robots’ sensing capabilities (e.g., vision, touch and hearing) to understand the human actions, and on robots’ physical capabilities (e.g., motion and speech) to physically interact with the human [1]. This interaction can be performed at different levels, from a completely distant interaction relying only on speech [2], to a close-contact interaction between the human and robot bodies [3]. A such close-contact interaction—generally referred to as physical human–robot interaction (pHRI)—is necessary for several applications, including human–robot collaboration with industrial robot arms [3], robot teleoperation [4], or robot-supported caregiving [5]. Compared to distant interaction, close interaction is harder to achieve, as it raises safety issues for both human and robot bodies.

One way to improve the interaction and particularly its safety is to use adequate sensors to provide information on the contact between the robot and the human [1] and then to use this information in an adequate controller. Suitable sensors for this application are “tactile skins”, i.e., tactile sensing arrays that can be integrated on robots such as the SonicSkin [6], or used as bionic skins for other applications, including healthcare [7]. In this paper, we use flexible tactile sensing arrays that can be mounted on any body part of a robot. This sensor acts as an array of force-sensing cells, where each cell can indicate the amount of force applied on its normal. Once acquired, the provided tactile information is processed and sent to our control framework, so that it can be used by a robot controller.

**Figure 1:**
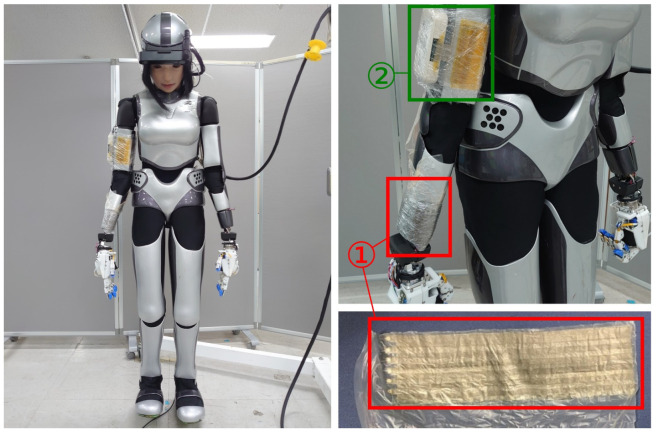
Photographs of HRP-4C humanoid robot with the flexible tactile sensor mounted on its right wrist and flattened sensing array: (1) flexible sensing array; (2) acquisition module sending data through Bluetooth.

Our goal is to develop tactile sensation for robots to realize safe human–robot physical interaction. We chose a humanoid robot HRP-4C [8] as a test platform, which we developed systems on. HRP-4C was designed as a “cybernetic human” capable of interacting distantly with humans using speech recognition; however, it is not designed to make physical contact. Hence, safety concerns remain for close physical interaction. To provide the sense of touch to this robot, we integrate a flexible tactile sensor as a tactile sensing skin, as shown in Figure 1, with the sensor attached to its right wrist. Our work contributes to the different components of the sensor integration, by showing that an end-to-end integration of the tactile sensor (including hardware integration, calibration, and software integration with the robot controller) can be performed on a humanoid robot. The use of this sensor for pHRI applications is a long-term goal of our research, as it first requires a complete integration of the flexible tactile sensor.

The main issue encountered with the integration of the tactile sensor is the method of finding correspondence between a point on the tactile sensor and a point on the robot. In this paper, we call this process a “spatial calibration”. Namely, in spatial calibration, we find the position and the normal direction of a point of each sensor cell in the robot frame. As explained in the next section, there is no existing work that targets this issue in the case of flexible sensors, except for simple surfaces (e.g., cylinder). In this work, we aim to enable spatial calibration on more complex rigid surfaces that can constitute body parts of humanoid robots. For this purpose, we develop a new method for spatial calibration. This method uses a triangular mesh as information on the surface covered by the sensor, and fits it with a B-spline surface. We test our method on the right wrist of HRP-4C, as it is an appropriate surface for pHRI experiments that is complex enough and can be fully covered by our tactile sensor. Then, our calibration is validated experimentally using two different methods. We discuss the performances and limitations of this method, including its precision and the type of surfaces that can be targeted. The development and validation of this new spatial calibration method is the main contribution of our work, as it enables the integration of flexible tactile sensors on complex robot’s surfaces, which was an unsolved problem in the existing literature. In this paper, we demonstrate that our method gives a good approximation of the 3D positions of the sensor cells and their normals, so that the sensor can be used in robot controllers.

## 2. Related Works

### 2.1. Sense of Touch for Humanoid Robots

Sense of touch is, with vision, the main sensing modality for a humanoid robot to interact with its environment [9]. Touch-enabled humanoid robots can be used in interaction scenarios such as object recognition or pHRI, as touch provides information on object’s shape, size, texture and movement, used to recognize an object or interpret a human’s intention [9,10]. Coupled with other perception mechanisms such as vision or hearing, touch can be useful in more complex scenarios as human–robot collaboration or teleoperation [4,11].

Sense of touch can be provided using tactile sensors, appropriately chosen with respect to the application. Different types of sensors have been proposed in the past, with varying sensing technologies and materials used. The four most common sensing mechanisms are piezoresistivity, piezocapacitivity, piezoelectricity and triboelectricity [9,10,12,13]. Less common mechanisms such as magnetic, barometric, optical, or impedance-based sensors have been developed on soft tactile sensing skins [9,13,14]. Sensors can also vary according to the materials used to take advantage of these mechanisms (polymers, electrodes) and to some design choices (number of layers, density, spatial orientation of the sensing elements) [10]. due to these various mechanisms, tactile sensors can provide force, pressure or proximity sensing. They can be coupled with other sensing capabilities such as temperature sensing or damage identification [14]. Some works proposed multimodal tactile sensors that merge several mechanisms [10,15].

Among the existing research on tactile sensors for robotic applications, some works have targeted the use of tactile sensing arrays comparable to that used in this work. They give interesting insights on the use of flexible tactile sensors for robotics and HRI. Among them, we can cite the works of G. Pang et al. [16], who developed a flexible tactile sensor for human–robot collaboration. The sensor is a flexible tactile array (similar to the one used in this study), and uses capacitive sensing. It has been integrated on YuMi robot arm, and aims to improve the safety of the pHRI. Also, a few works specifically target the integration of these sensors on humanoid robots [15], even though they use different modalities from the one we propose.

### 2.2. Flexible Tactile Skins

In our work, we target sensors that have a specific property: flexibility. This property has emerged with recent progress in material engineering and sensor design that lead to tactile sensors imitating the characteristics of the human skin: soft, thin, flexible, lightweight, and possibly stretchable [14]. The flexibility is particularly appreciated as it allows the sensors to be wrapped on most of the surfaces of the robot or the device it is integrated on [14,17]. This property enables the use of these sensors in several application fields, including health monitoring, medical diagnosis, artificial intelligence, and electronic skin for robots or enhanced humans [13,18].

Flexible tactile skins can be based on the different sensing mechanism listed previously. The flexibility can be achieved with the use of flexible electronics as flexible electrodes, nanomaterials (e.g., carbon nanotubes, nanofibers), and flexible composite materials for the different layers of the sensor [13]. In recent works, researchers achieved to create flexible, thin, human-like tactile skins without sacrificing efficient sensing, i.e., high sensing range, spatial precision, sensitivity, and low Young’s modulus [13,14,19].

The recent progress in flexible tactile skins includes the creation of ultrathin sensors with good sensing capabilities [19], the use of new materials such as conjugated polymers [18] or Parylene MEMS [20], the use of new sensing modalities as electrical impedance tomography [17], the development of skins with damage detection capabilities [21], the reproduction of complex human-like sensing imitating the fingertips [20], integration on robots [16], and combined use with artificial intelligence methods as deep learning [22]. As we can see, progress is made on both materials side and application side, including robotics applications. These progress constitute significant advances in the integration of flexible tactile sensors as tactile skin for humanoids robots. However, sensor spatial calibration is not the main concern of these recent works.

### 2.3. Tactile Sensor Calibration

The main issue encountered with the integration of the tactile sensor is the one of its spatial calibration, i.e., finding the position of each sensor cell and the direction of the applied forces in the robot frame. This issue is not targeted in most of the works cited above. For non-flexible sensors, some calibration methods have been proposed, as automatic spatial calibration using 3D reconstruction and pose estimation [15], or calibration based on self-contact of the humanoid robot on its skin [23]. In the case of flexible sensors, it may be assumed that the surface is simple enough to make the calibration easy (e.g., cylinder), as in [16]; generally the spatial calibration is not even mentioned.

In our work, we want to enable spatial calibration of flexible sensors on a wide range of robot surfaces; thus, it is important to consider more complex shapes. As in the previously mentioned works, it is suitable to have a parametric representation of the covered surface to calibrate a sensor. To the best of our knowledge, no work has been specifically performed on a generic solution to calibrate a flexible tactile sensor on any surface. However, it is easy to obtain triangular meshes of the supporting surface (they are generally given by robot manufacturers or can be obtained with 3D scan), and the problem of finding the parametric expression of a surface from a given triangular mesh or point cloud has been widely studied in the field of computer graphics. Several approaches have been proposed in the past. The most common approach is to use least squares approximation to fit a parametric surface, e.g., a B-spline, to the given mesh [24]. This approach gives good results for surfaces that are flat enough; however, it is not adapted for more complex surfaces such as the body parts of a humanoid robot, e.g., the right wrist of HRP-4C, which has an irregular cylinder-like shape. Other algorithms have also been experimented on, for example genetic algorithms [25] or iterative algorithms [26], but they appear to have the same limitations. For more complex surfaces, several approaches specific to certain shapes have been proposed. For example, the approach of X. Liu et al. is adapted to surfaces of revolution [27], while the one of S. Wang et al. targets high-speed train heads [28]. More recently, some methods have been developed to fit a mesh of any shape to a parametric surface, such as deep neural networks that learned the surface-fitting operation from a large dataset of meshes [29,30,31]. These methods—especially [29]—show good results for meshes of any shape, but the obtained surface is hard to adjust to the needs of the user. These limitations motivated the development of a new method for spatial calibration.

## 3. Materials and Methods

### 3.1. Flexible Tactile Sensor

The tactile sensor we use is the stretchable flexible sensing array we developed previously at the Human Augmentation Research Center (HARC), AIST [32]. It is a flexible sheet that can be applied to any soft or rigid surface. Its sensing surface is an array of 6 × 22 sensing cells of 1 cm${}^{2}$ each, able to detect the normal force applied to the cell. In the present work, the sensor is used in its initial, non-stretched configuration.

Sensor cells are formed by flexible wiring/electrodes attached to an adhesive layer, and elastic films. Thus, they can be folded in any direction [32]. The flexible sensor has been covered with plastic to fix it to the robot’s wrist without altering the sensor. It is connected to an acquisition module used to interface the sensor with a robot, as displayed in Figure 1. The tactile sensor provides information about the pressure applied to the direction of the normals to each cell, which can be in any direction due to the flexible shape of the sensor. The chosen hardware for this tactile sensor uses capacitive sensing and provides two sensing ranges: one for low pressures with high sensitivity (10 dgt./kPa), and one for high pressures with a limit of detection of 200 kPa. Additional details on the sensor physical properties and performances are given in Table 1, and the output curves of the sensor are given in Figure S1.

As it is based on capacitive sensing, the sensor detects the changes of its internal capacitance $C_{k,l}$ for each cell (k,l)∈[[0,6]]×[[0,22]] that is numerically output as an intensity $I_{k,l}$. This intensity is related to the norm $F_{k,l}$ of the force applied along the normal to the cell. To be able to use the tactile sensor on our robotic systems, we calibrate our sensor by interpolating the polynomial relation $I_{k,l}=f\left(F_{k,l}\right)$ from a set of measurements.

### 3.2. Tactile Information Framework

**Figure 2:**
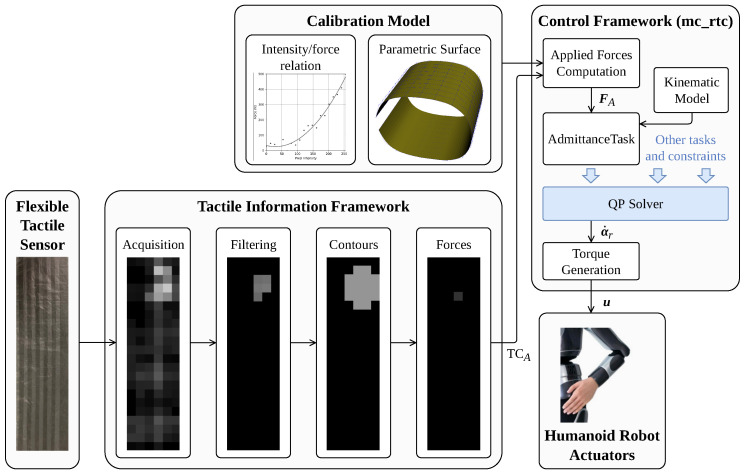
Simplified diagram of humanoid robot control from tactile sensor information. Sensor data are acquired and processed with the tactile information framework and are used in the mc_rtc-based controller. The displayed controller uses admittance control, but other control tasks can be used.

A software framework has been developed to handle the information from the tactile sensor to be able to use it in our robot controllers. This tactile information framework is designed as a package for the Robot Operating System (ROS), melodic version [33]. It acquires the data from the sensor using Bluetooth Serial Port Profile (SPP), then applies different computations to obtain the forces applied to the normal to the cells, and finally sends them as a control input to our robot controller based on mc_rtc (version 1.14), an interface for simulation and robot control systems [34]. An overview of the whole process with the tactile information framework and the control framework is given in Figure 2.

The tactile information framework acquires array-like data that contain a pressure intensity encoded in 8 bits (from 0 to 255) for each cell (k,l). This data format is equivalent to a monochrome image of 6 × 22 pixels, with the pressure intensity corresponding to the value of a pixel. These similarities between tactile and image data have already been exploited in previous works [35,36]. Thus, the framework uses OpenCV [37] library (version 3.1) and embeds common computer vision algorithms. Four processes are successively used to compute the applied forces from the raw sensor data, as shown in Figure 2, namely acquisition, filtering, contour detection, and forces computation. These processes are applied for each time *t*.

The first of these processes is the acquisition. It reads the raw data using the Serial [38] library (version 1.1) and transforms it into image-like data. For performance considerations, the acquisition algorithm runs in a thread separated from the other processes.

The second process is the filtering. As displayed in Figure 2, the raw data acquired by the sensor is very noisy. The filtering step aims to remove this noise before the data are processed. The objective is to keep only the areas where pressure is applied. To achieve this, we apply an adaptive threshold filter [39] that acts on the image as a binary mask. Compared to a simple threshold filter, the threshold value is computed at the neighborhood of each pixel instead of being constant.

The next step is to identify the areas where there is tactile contact; it is performed in the contour detection process. This process uses a common contour detection algorithm [40] to detect the contours of the different zones of the image with non-null pixels. After this step, we dispose of distinct areas $A\in [\;\left[0,n_{\mathrm{areas}}\right]\;]$ that correspond to the different areas where contacts are applied to the sensor, for each time *t*.

The last process, referred to as forces computation, aims to compute the distinct pixels where forces are applied to the sensor. We consider that for each area *A*, the force contact is applied to the centroid $c_{A}$, weighted with the intensity of the pixels inside the contour:

(1) $$c_{A}=\frac{1}{\#A}\sum_{(k,l)\in A}I_{k,l}\left(\begin{array}{c} k\\ l \end{array}\right)\;,$$

where #A is the number of pixels (k,l) in the area *A*.

At the end of these processes, the tactile information framework outputs a list of custom ROS messages named tactile contact (TC), each of them composed of the position of the centroid where the force is applied, and the sum of the pressure intensities in the corresponding area:

(2) $${\mathrm{TC}}_{A}=\left[k_{A}\;,\;l_{A}\;,\;\sum_{(k,l)\in A}I_{k,l}\right]\;s.t.\;{\left(k_{A},l_{A}\right)}^{T}=c_{A}\;.$$

This message is sent for each area A(t) for each time *t*. The message is then used in the control framework as explained in the next paragraph.

### 3.3. Control Framework

The control framework used is mc_rtc [34], which provides a bridge with ROS for the communication with the tactile information framework. It handles full-body control of several humanoid and non-humanoid robots, including HRP-4C. It embeds several control tasks based on quadratic programming (QP), including position control of end-effectors, posture control, admittance control and other control tasks.

We created a mc_rtc package for tactile-based control that interfaces with the tactile information framework, implemented in C++. It obtains the TC messages from the framework and uses the calibration model of the tactile sensor to compute the actual forces applied to the wrist of the robot in its own frame. The calibration model is composed of two parts, as shown in Figure 2:

- The intensity/force relation, which is the relation between the intensity obtained with the acquisition software and the actual force applied. This relation is interpolated from experimental data.
- The spatial calibration model, which is the expression of the surface of the sensor as a B-spline surface. The method used to obtain the B-spline expression is detailed in the next subsection.

From this calibration model and the obtained messages ${\mathrm{TC}}_{A}$, our package computes the applied forces $F_{A}$ for each time *t*. These forces can be used as input for a control task, for example the admittance task [41].

### 3.4. Spatial Calibration

#### 3.4.1. Overview

**Figure 3:**
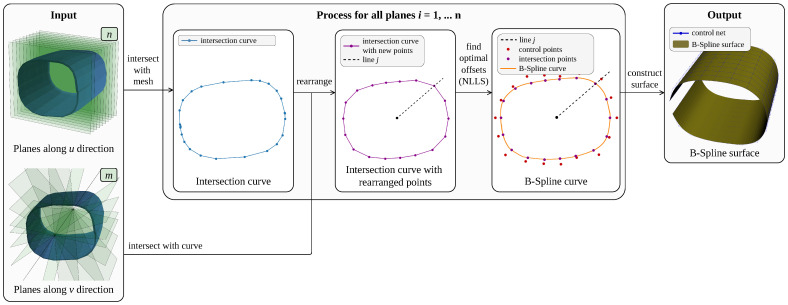
Construction process of the B-spline surface. From a given mesh and sets of planes along two distinct directions, the process generates a B-spline surface that fits the original mesh. The distances between the intersection points and the control points are exaggerated on the figure for better visualization.

In this subsection, we detail the method we developed for the spatial calibration of the tactile sensor from a given triangular mesh. This method aims to be used with any mesh of a robot part and is tested on HRP-4C’s right wrist. It is based on non-linear least squares (NLLS) fitting with additional geometric constraints that allow it to be used on non-flat surfaces. As stated previously, the sensor surface is assumed not to be stretched. An overview of the whole process is given in Figure 3.

The algorithm is implemented as a Python package. It relies on Trimesh (version 3.15) [42] to handle triangular meshes, NURBS-Python (version 5.0) [43] to handle B-spline curves and surfaces, and common scientific Python libraries.

In this work, we manipulate B-spline objects, which can be defined as piecewise polynomial splines (curves or surfaces) that have minimal support with respect to a given degree, smoothness, and domain [44]. We also acknowledge that Non-Uniform Rational B-splines (NURBS) are widely used in computer graphics applications and could be used in our calibration method instead of B-splines. These splines can be defined as weighted B-splines [44], so that using them will add parameters that would not be used in our fitting method as explained in Section 3.4.3.

#### 3.4.2. Inputs

The main input provided by the user is the triangular mesh that the surface should cover, as close as possible from the real surface, with the exact dimensions. This mesh can be given by the robot manufacturer or obtained with a 3D scan. If the surface is not fully covered by the sensor, its mesh should first be reduced to the covered part, for example using mesh cutting tools [45]. The user should also provide the dimensions of the tactile sensor and the point on the mesh that corresponds to the origin of the sensor.

In addition, the user should provide a 3D grid, i.e., two sets of planes, each along different directions. This 3D grid should separate the mesh into n+1 portions along a first direction (that corresponds to the *u* parameter of the B-spline) and m+1 portions along a second direction (*v* parameter). The definition of these planes is dependent of the geometry of the surface we are considering, so that there is no generic method to obtain them; what matters is to have the desired number of sections along the two directions (these numbers *n* and *m* can be adjusted by the user) and sections of similar length. In our case, the right wrist of HRP-4C has a cylinder-like shape; thus, we decided to define a first set of planes perpendicular to the main axis (*u* direction), and a second set of planes intersecting on this main axis and separated from a constant angle (*v* direction). An overview of these planes is given in the Input section of Figure 3.

#### 3.4.3. Process

The process to construct the B-spline is an iterative process for all planes $P_{u}^{i},i\in \left[\;\left[1,n\right]\;\right]$. The details of the process are given in Algorithm 1. It first computes the intersection between the mesh and the plane $P_{u}^{i}$. This intersection is a curve formed by a set of ordered points (non-parametric). As these points are not evenly separated, the algorithm then computes the intersection between this curve and all the planes $P_{v}^{j},j\in \left[\;\left[1,m\right]\;\right]$ along *v* direction. This results in a similar curve but with all the separation points lying on the planes along *v* direction $P_{v}^{j}$. The obtained curve is projected to a 2D space and then approximated with a B-spline curve using constrained NLLS. Commonly, NLLS algorithms use the set of points to find the optimal control points that define the B-spline curve [44], i.e., solving the following optimization problem:

(3) $${\left(P_{1,\mathrm{opt}},\cdots P_{N,\mathrm{opt}}\right)}^{T}=\underset{\left(P_{1},\cdots P_{N}\right)\in R^{2\times N}}{arg min}\sum_{k=1}^{M-1}\left({\left|Q_{k}-\sum_{i=0}^{N}N_{i,p}\left(v\right)P_{i}\left(v\right)\right|}^{2}\right)\;.$$

where *N* is the number of control points that should be found, *M* the numbers of points in the set of points, $Q_{k}$ the points in the set of points, and the sum inside the square norm the B-spline curve C(v). It is assumed that $C\left(0\right)=Q_{0}$ and $C\left(1\right)=Q_{M}$.

In our case, we constrain the NLLS algorithm so that the control points lie on the lines of intersection between the plane $P_{u}^{i}$ and the planes $P_{v}^{j}$. Thus, the control points are defined by an offset distance that separates them from the corresponding points $Q_{k}=Q_{j},j\in \left[\;\left[1,n\right]\;\right]$. In that case, the optimization problem can be reformulated as:

(4) $${\left(P_{1,\mathrm{opt}},\cdots P_{m,\mathrm{opt}}\right)}^{T}=\underset{P_{1}\in L_{1},\cdots P_{m}\in L_{m}}{arg min}\sum_{k=1}^{m-1}\left({\left|Q_{k}-\sum_{i=0}^{m}N_{i,p}\left(v\right)P_{i}\left(v\right)\right|}^{2}\right)\;.$$

where $Q_{k}$ are the points in the set of points, $L_{j}$ the lines of intersection between the plane $P_{u}^{i}$ and the planes $P_{v}^{j}$, and $N_{i,p}\left(v\right)$ the B-spline basis functions generated with uniform knots. It is also assumed that $C\left(0\right)=Q_{0}$ and $C\left(1\right)=Q_{M}$. It appears that there is *m* control points to find.

Due to this constraint, the control points are aligned in both *u* and *v* directions, so that they can be used to reconstruct a B-spline surface. Without this constraint, the reconstructed B-spline surface would have many wiggles due to the misalignment of the control points. After the computation of the control points, the obtained curve is projected back to a 3D space. All the curves are then used to construct the B-spline surface.
**Algorithm** **1.** Construction of the B-spline surface from a mesh and a tridimensional grid.**Input:** Integer k>1, mesh M, lists of planes $P_{u}$ and $P_{v}$ along *u* and *v* directions
**Output:** B-spline surface S  1: $C\leftarrow [{\left[\right]}_{1},{\left[\right]}_{2},...,{\left[\right]}_{n}]$▹ List of B-spline curves  2: $n\leftarrow \mathrm{Len}(P_{u})$  3: $m\leftarrow \mathrm{Len}(P_{v})$  4: **for**
i∈[1,n]
**do**  5:   $C_{i}\leftarrow \mathrm{MeshPlaneIntersection}\left(M,P_{u}\left[i\right]\right)$▹ List of curves  6:   $C_{i,2D}\leftarrow \mathrm{Project}2D\left(C_{i},P_{u}\left[i\right]\right)$▹ List of curves (2D)  7:   $L_{i,2D}\leftarrow \left[{\left[\right]}_{1},{\left[\right]}_{2},...,{\left[\right]}_{m}\right]$▹ List of lines (2D)  8:   $IP_{i,2D}\leftarrow \left[{\left[\right]}_{1},{\left[\right]}_{2},...,{\left[\right]}_{m}\right]$▹ List of intersection points (2D)  9:   $CP_{i,2D}\leftarrow \left[{\left[\right]}_{1},{\left[\right]}_{2},...,{\left[\right]}_{m}\right]$▹ List of control points (2D)10:   $CP_{i}\leftarrow \left[{\left[\right]}_{1},{\left[\right]}_{2},...,{\left[\right]}_{m}\right]$▹ List of control points11:   **for** j∈[1,m] **do**12:    $L_{i,2D}\left[j\right]\leftarrow \mathrm{PlanePlaneIntersection}\left(P_{v}\left[j\right],P_{u}\left[i\right]\right)$13:    $IP_{i,2D}\left[j\right]\leftarrow \mathrm{CurveLineIntersection}\left(C_{i,2D},L_{i,2D}\left[j\right]\right)$14:    $CP_{i,2D}\left[j\right]\leftarrow \mathrm{OptimalControlPoints}\left(IP_{i,2D}\left[j\right],L_{i,2D}\left[j\right],C_{i,2D}\right)$15:    $CP_{i}\left[j\right]\leftarrow \mathrm{Project}3D\left(CP_{i,2D}\left[j\right],P_{u}\left[i\right]\right)$16:   **end for**17:   $C\left[i\right]\leftarrow \mathrm{BSplineCurveFromControlPoints}\left(CP_{i}\right)$18: **end for**19: S←BSplineSurfaceFromCurves(C)

#### 3.4.4. Output

The B-spline surface S(u,v) is generated using the obtained B-spline curves. It uses the same control points and degree. Knot vectors *U* and *V* along *u* and *v* directions are generated as uniform knot vectors. Finding optimal knots would add a dimension to the search space of our NLLS algorithm and exponentially increase its complexity. Similarly, finding optimal weights would have the same effect on the NLLS algorithm; this is the reason why we use non-weighted B-splines instead of NURBS.

The obtained B-spline surface is the output of our implementation of the calibration method. It is an approximation of the surface of the robot mesh but can be easily adjusted to the sensor dimensions [46] if it does not cover the full mesh.

The obtained B-spline surface can easily be used to compute the positions of each cell of the wrapped tactile sensor and its normal vector:

- The position of a cell (k,l)∈[[0,6]]×[[0,22]] is automatically given by evaluating the B-spline surface $S(u_{0},v_{0})$ at parameters $u_{0}=k/6$ and $v_{0}=l/22$.
- The normal vector to a cell (k,l) can be computed using partial derivatives of the B-spline surface as:
  (5) $$n\left(u_{0},v_{0}\right)=r_{u_{0}}\times r_{u_{0}}=\left(\begin{array}{c} \frac{\partial x}{\partial u}\left(u_{0},v_{0}\right)\\ \frac{\partial y}{\partial u}\left(u_{0},v_{0}\right)\\ \frac{\partial z}{\partial u}\left(u_{0},v_{0}\right) \end{array}\right)\times \left(\begin{array}{c} \frac{\partial x}{\partial v}\left(u_{0},v_{0}\right)\\ \frac{\partial y}{\partial v}\left(u_{0},v_{0}\right)\\ \frac{\partial z}{\partial v}\left(u_{0},v_{0}\right) \end{array}\right)\;,$$

where $u_{0}=k/6$ and $v_{0}=l/22$.

Those computations are implemented in the control framework to compute normals and positions in real time using sensor data.

### 3.5. Validation Experiment

#### 3.5.1. Setup

We designed an experiment to validate the spatial calibration method. It consists in touching the tactile sensor mounted on the robot wrist at different points. Two methods are used to compute the positions and directions of the applied contacts: one using a force/torque sensor (FTS) and another using a motion capture system. A photograph of the elements of the setup is given in Figure S2. The results obtained with these methods are used as ground truth for the spatial calibration method.

We make use of the following setup:

- A support where an ATI Mini58 FTS, the robot right wrist, the tactile sensor and motion capture markers are mounted around the wrist. The relative positions of all these elements are known.
- A metal wand used to touch the tactile sensor, also with motion capture markers with known relative positions.
- A motion capture system composed of 10 Optitrack Prime 13 cameras.

**Figure 4:**
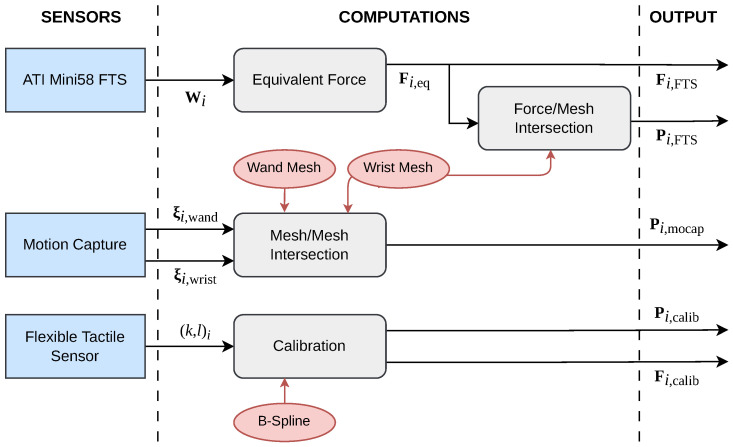
Flowchart of the experimental process. The data from the sensors (in blue) and the geometric objects (in red) are used to compute the applied forces $F_{i}$ and contact points $P_{i}$ through different methods.

The data from the FTS, the tactile sensor, and the motion capture are captured through ROS and are used to compute the force vectors applied to the tactile sensor and the contact points, as shown in the flowchart in Figure 4. The computations are implemented in Python under a ROS package and are detailed in the following paragraphs. As there is a finite number of touches performed during the experiment, we will denote $i\in [\;\left[0,N_{\mathrm{touch}}\right]\;]$ as the index of the touch.

The two methods do not play the same role in the validation experiment. The FTS was chosen, as it can be easily attached to our surface and provide accurate information on the applied contact (position and direction) by computing the equivalent force, as explained in the next paragraph. On the other side, the motion capture system can be used only to compute the position of the contacts, not their directions. As it has a lower precision compared to the FTS, its main aim is to provide a second validation and to check the coherency of the obtained results.

#### 3.5.2. Using Force/Torque Sensor

The FTS provides the force ${\left\{F_{i}\right\}}_{0}$ and torque ${\left\{N_{i}\right\}}_{0}$ applied when touching the tactile sensor, expressed in its own frame $R_{0}=\left(O;x_{0},y_{0},z_{0}\right)$. As the force and couple are perpendicular, this force/couple system $W_{i}=\left\{F_{i}\;N_{i}\right\}$ is equivalent to another force/couple system $W_{i,\mathrm{eq}}=\left\{F_{i,\mathrm{eq}}\;0\right\}$ with a null moment. This new system can be obtained by moving the resultant force $F_{i}$ of a distance $d_{i}$ along the line perpendicular to the plane of the resultant force $F_{i}$ and resultant couple $N_{i}$ until the resultant force creates a moment equivalent to $N_{i}$.

**Figure 5:**
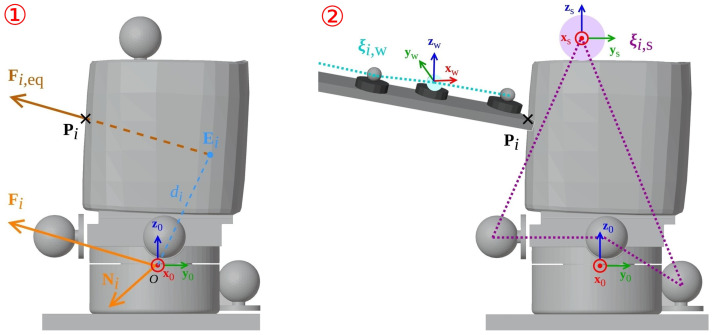
Schematic of the estimation of the pressure points $P_{i}$ during the experiment using two methods: (1) Using equivalent forces: the equivalent force $F_{i,\mathrm{eq}}$ is computed geometrically from the resultant force and torque. (2) Using motion capture: the motion capture system gives the relative poses of the wand and the support, whose respective meshes intersect at the pressure points.

The equivalent force $F_{i,\mathrm{eq}}$ can be found using a geometric method, as shown in Figure 5. $F_{i,\mathrm{eq}}$ is defined by its direction, which is the same as $F_{i}$, and its origin $E_{i}$ that can be found geometrically as:

(6) $$E_{i}=d_{i}\left(\frac{F_{i}\times N_{i}}{∥F_{i}\times N_{i}∥}\right)\;.$$

The distance $d_{i}$ present in this expression can also be computed from the resultant force $F_{i}$ and torque $N_{i}$. Let us separate $N_{i}$ into two components: $N_{i}=N_{i,\|}+N_{i,⊥}$, such that $N_{i,\|}$ is in the same direction as $F_{i}$ and $N_{i,⊥}$ is orthogonal. We denote θ as the angle between $N_{i}$ and $N_{i,\|}$. It results as

(7) $$d_{i}=\frac{∥N_{i,⊥}∥}{∥F_{i}∥}\;,$$

where

(8) $$\begin{array}{cc} N_{i,⊥} & =N_{i}-N_{i,\|}\\ \; & =N_{i}-∥N_{i}∥\cos\theta \left(\frac{F_{i}}{∥F_{i}∥}\right)\\ \; & =N_{i}-∥N_{i}∥\frac{N_{i}\;\cdot \;F_{i}}{∥N_{i}∥∥F_{i}∥}\left(\frac{F_{i}}{∥F_{i}∥}\right)\\ \; & =N_{i}-\frac{N_{i}\;\cdot \;F_{i}}{{∥F_{i}∥}^{2}}\;F_{i}\;. \end{array}$$

Finally, $E_{i}$ can be expressed as:

(9) $$E_{i}=\frac{1}{∥F_{i}∥}∥N_{i}-\frac{N_{i}\;\cdot \;F_{i}}{{∥F_{i}∥}^{2}}\;F_{i}∥\left(\frac{F_{i}\times N_{i}}{∥F_{i}\times N_{i}∥}\right)\;.$$

The obtained force vector $F_{i,\mathrm{eq}}$ goes through $E_{i}$ and corresponds to the force vector applied by the wand to the tactile sensor. Thus, it intersects with the wrist and the tactile sensor on a point $P_{i}$, as displayed in Figure 5. The equivalent force vector $F_{i,\mathrm{eq}}$ can be compared to the normal vector obtained with the calibration method (see Equation (5)), and the intersection point $P_{i}$ can be compared to the position of the cell obtained with the calibration method.

#### 3.5.3. Using Motion Capture

The motion capture system provides the poses of the support ${\left\{\xi_{i,s}\right\}}_{0}$ and the wand ${\left\{\xi_{i,w}\right\}}_{0}$ expressed in the world frame, which is the FTS frame $R_{0}$, as shown in Figure 5.

Using these data, we can compute for each touch *i* the intersection between the wand mesh and the wrist mesh, using mesh intersection algorithm [47]. The resulting intersection can be expressed as a set of points $P_{k,i}$. We use the centroid $P_{i}$ of this set of points for comparison with the sensor cell position.

Contrary to the method using FTS, this method does not allow for computing the direction of the force vector. It aims to validate the position of the sensor cell with another method.

## 4. Results

### 4.1. B-Spline Surface Generation

B-spline surfaces of the right wrist of the HRP-4C robot are generated using the calibration method presented in Section 3.4, with varying parameters *n* and *m*. In the input section of Figure 3, the two sets of planes used for the generation of the B-spline are shown, in the case of n=m=10. Both of them are constructed depending on the geometry of the mesh to fit. In our case, the right wrist mesh has a cylinder-like shape around a main axis *L*, which can be defined by hand by joining the centroids of two sections of the mesh. This axis is used to define the separation planes as follows:

- The planes along *u* direction $P_{u}$ are perpendicular to the main axis *L* and are separated with a constant distance
  (10) $$d=\frac{d_{L}-2d_{\mathrm{offset}}}{n}\;,$$
  where $d_{L}$ is the length of axis *L* crossing the convex hull on the mesh and $d_{\mathrm{offset}}$ a distance offset added to ensure that all the plane sections are complete.
- The planes along *v* direction $P_{v}$ cross on axis *L* and are separated with a constant angle
  (11) $$\alpha =\frac{\pi }{m}\;.$$
  By construction, they are orthogonal to the planes $P_{u}$.

To evaluate the precision of the fitting algorithm, we computed the distance between the obtained B-spline and the original right wrist mesh. Table 2 shows this difference with varying parameters *n* and *m*, in addition to the time required to generate the B-splines. Knot vectors are automatically generated as uniform knot vectors, as explained in Section 3, and smoothing factor is set to 0.01 for all the splines. The distances were computed using Meshlab [48] software (version 2022.02). B-splines were generated on a standard laptop PC with 12-core Intel Core i7-10750H 2.60GHz CPU.

As we can see, the obtained B-spline is close to the original mesh, with a mean distance of 0.854 mm (geometric) or 0.885 mm (Hausdorff) for n=m=10. The mean distances decrease slightly when we increase the numbers of separating planes *n* and *m*, as it adds more points to the NLLS algorithm. Because of the geometry of the mesh, we see that the parameter *n* has more impact on the mean error than *m*. At the same time, it is observed that the parameter *m* has more impact on the maximal distance than the mean one. This particularity can be explained by the fact that the curvature is not the same on all parts of the mesh. As the planes are evenly separated, a reduction of the numbers of planes can lead to areas with high curvature and very few points to interpolate, which lead to important errors in the NLLS approximation. This is particularly visible with the reduction of the parameter *m*, as the surface has more curve in the *v* direction.

### 4.2. Validation of Experimental Method

Before going through the validation of the spatial calibration method, we validate with simulation the theoretical analysis developed in Section 3.5. In particular, we test the geometric method used to compute the applied contact from FTS data using equivalent forces. We recall that a force/couple system with perpendicular force and couple can be reduced to an equivalent system with a force and no couple, by moving the resultant force on a line perpendicular to the system’s coplanar plane until the couple is null [49]. To verify our method with respect to this definition, we simulate 10,000 randomly generated force/couple systems with perpendicular force and couple with the Python script given in Algorithm S1. For each generated system, we compare the application point of the equivalent force vector obtained with the definition and the one obtained with our geometric method (see Equation (9)). A visualization of the sorted absolute direction errors between the two methods is given in Figure S3. We can observe that the error is always between 0.0 and 2 × 10${}^{-16}$ on normalized vectors, with a mean of 6 × 10${}^{-20}$, so that we can validate our geometric method. The discrete aspect of Figure S3 and the low values of the obtained errors show that the source of error is either numerical approximation or optimization residuals and not the inaccuracy of the tested method.

The second experimental method using motion capture has been tested by manually setting poses for the wand and wrist meshes. The mesh poses and the intersection point (if any) have been visualized with ROS Rviz, and we observed coherent results. As a single test is not sufficient, the same visualization has been checked during the real-time experiment and showed coherent positions of the intersection points.

### 4.3. Validation of Spatial Calibration Method

#### 4.3.1. Experimental Data

The touch experiment presented in Section 3.5 was performed with 19 distinct touches of the tactile sensor at different points. Among them, 11 resulted in non-null mesh intersections from the motion capture system and non-null force/mesh intersections with the equivalent forces. The computations of the contact positions and normals are performed in real time, as they require only the few computations presented in Section 3.4.4.

**Figure 6:**
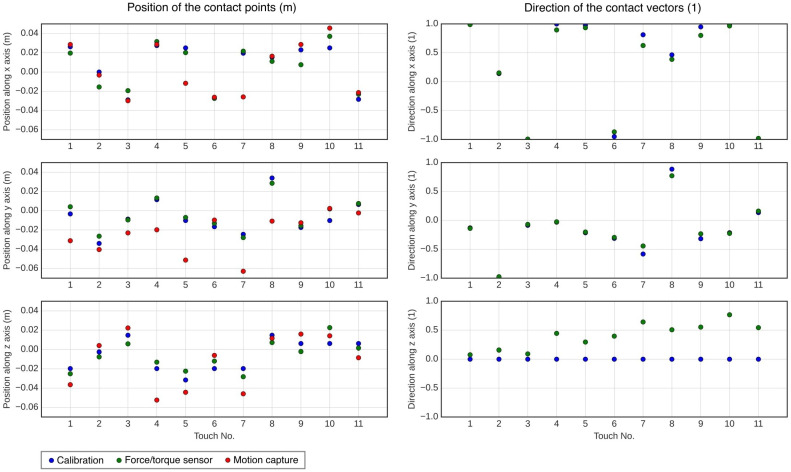
Visualization of the positions and directions obtained during the experiment. Exact values are given in Table S1.

Figure 6 shows the direction and intersection results obtained for these 11 touches and the corresponding results obtained with the calibration method. The exact values are given in Table S1. The B-spline surface used for comparison has been generated with parameters n=10 and m=10.

In the given results, the applied forces are defined with their origin and directions. As all of them intersect with the mesh, we consider the intersection points as the origins of the force vectors. Thus, if we compare the positions of the intersection points and the directions of the vectors, we are able to validate, or not, the positions and normals given by the calibration method.

To be able to compare the obtained directions and positions, some transformations are required. To compare the directions, we divide the force vector by their norms to obtain comparable unit vectors. To compare the positions, we express them in the experiment frame $R_{\exp}$, which is equal to the FTS frame $R_{0}$ plus a translation along *z*. The transformations from the motion capture frame and the FTS frame to the experiment frame are found manually from the dimensions of the wrist support, which are known. The data displayed in Figure 6 and Table S1 are given in the experiment frame $R_{\exp}$.

In addition, to avoid high variations during the measurements, we applied low-pass filters to the raw FTS data $\left\{F_{i}\;N_{i}\right\}$ and to the pose of the support $\xi_{i,s}$.

#### 4.3.2. Validation of the Normals to the Cells

**Figure 7:**
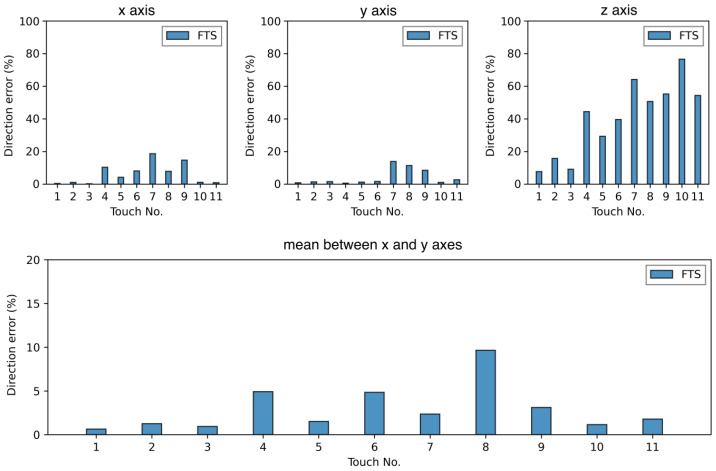
Absolute error between the direction vectors obtained with the force/torque sensor (FTS) and the calibration method. The vectors are normalized before comparison, so that the results are unitless. We can observe much higher errors along the *z* axis.

Firstly, we compared the normals to the cells given by the calibration method and those obtained from the FTS. Figure 7 shows the absolute values of the difference between the vector directions for *x*, *y* and *z* axes, computed directly from the data of Table S1. We observed that the mean error on the *z* axis that almost coincides with the main axis *L* after transformation to the experiment frame is high for each touch, with a mean of 40.6%.

We made the hypothesis that the error is caused by an abnormal value of the force along *z* given by the FTS. A possible explanation of this phenomenon is that the FTS we use has a lower resolution on the *z* axis than on the *x* and *y* axes: for the configuration, we used (SI-1400-60), and the force sensing resolution was 7–12 N for the *z* axis and 1–3 N on *x* and *y* [50]. This lower precision, combined with the high sensing range of the sensor, could lead to unstable results if the force applied is too low, as was the case when the FTS was touched with the wand (applied forces were between 1 and 16 N along the *z* axis, with a mean of 3.6 N). Less probable, this error might result from a wrong force calibration of the FTS along *z*. The use of a second sensing method—in our case the motion capture—aims to verify the coherence between the results obtained and to validate, or not, the hypothesis made on the FTS. This will be discussed in the next section.

The last plot of Figure 7 shows the error between the vector directions but that which is limited to *x* and *y* coordinates, to correct the data after this hypothesis. As we can see, the errors are much lower if we do not consider the *z* axis. In that case, the directions given by the FTS and those given by the calibration method are close, with a mean error of 2.9%. This low error validates that the calibration method gives good directions for the normals to the cells in the *x* and *y* directions.

#### 4.3.3. Validation of the Cell Positions

**Figure 8:**
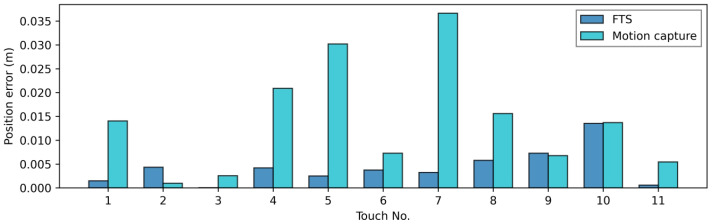
Mean position error on intersection points, in m. Errors are computed between the validation method—force/torque sensor (FTS) or motion capture—and the calibration method. The FTS is assumed to give exact *z* values for the force.

Secondly, we compared the positions of the cells given by the calibration method and those obtained with the other methods. Figure 8 presents the mean position error on the intersection points. In this figure, the hypothesis on *z* values stated in the previous paragraph is considered as true (as in Table S1), such that the error on *z* is reduced to 0 before plotting.

As we can observe, the position error obtained by computing the equivalent force from FTS data gives a position that fits the one given by the calibration, with a mean error of 4.2 mm, which is lower than the dimension of a cell ( 1 cm× 1 cm), and a maximum error of 1.35 cm.

The position error given by the motion capture is much higher, with a mean of 1.40 cm and a maximum of 3.36 cm, but the positions given are also coherent with the ones obtained with the calibration. According to Optitrack documentation [51], the typical measurement error is less than 0.2 mm. However, the error can increase if there are too many occlusions of markers that prevent the motion capture system from perfectly reconstructing the solids. In our case, the wand and the support only have four motion capture markers each, and such occlusions sometimes append on the support’s markers during the experiment. Furthermore, motion capture markers are close because of the size of our setup, which results in lower precision of the motion capture system. Thus, we can consider that the position error is mostly due to the motion capture system.

## 5. Discussion

In this paper, we developed a new calibration method that is able to approximate a 3D mesh with a B-spline surface. This new method can be easily parameterized depending on the separation planes chosen by the user. Due to this manual parameterization, the method is not limited to flat surfaces as common NLLS approximation methods, and it can be used on complex surfaces such as the body parts of a humanoid robot. The generation of the B-spline surface has been tested on the mesh of the right wrist of a HRP-4C humanoid robot. It shows approximation errors similar to what can be obtained with common NLLS approximation [24] or deep-learning-based methods [29,30,31]. It has been observed that the error is higher where the mesh has higher curvature. This is due to the fact that the method has been tested with evenly separated planes. In the case of HRP-4C’s wrist, there is no section with strong curvature; thus, the approximation is still valid. For meshes with higher curvatures or edges, the method has not been validated. Consequently, it is currently limited to smooth surfaces. However, the method can be easily adjusted, and adding more separating planes can improve its accuracy, as shown in Section 4.1. The possibility of such adjustments is one of the main advantages of the designed method.

Then, the generated B-spline surface is integrated into the humanoid robot model to locate the positions and directions of the contacts applied to the robot. An experiment has been performed to validate the positions and directions of the contacts with two methods. The first method consisted in computing the force applied to the wrist from the force and torque of an attached FTS. During the experiment, it appeared that the *z* values given by the FTS were abnormally high, and we made the hypothesis that the source of error was the FTS. A possible explanation for the source of error being the FTS is its lower resolution for forces along the *z* axis, which can lead to unstable results, as the applied forces were low. By not considering the error on the *z* axis, it appeared that the results on *x* and *y* were coherent. At the end, this method could validate that the positions and directions obtained on the B-spline surface are valid, with a mean error of 3% for the directions and 4.2 mm for the positions, which is similar to the size of one cell (1 cm × 1 cm). The positions of the cells have also been validated through a second method that estimates the contact as the intersection between the wand and wrist meshes, whose poses were captured with motion capture. The result given by this method are coherent with the one obtained previously, with a higher mean error of 1.40 cm. We think this lower precision of the method using motion capture can be explained by well-known occlusions issues, which are coherent with the low number of markers used during the experiment (four for each object) and their proximity. As the results obtained were coherent with the spatial calibration along all axes, and with the FTS experiment for the *x* and *y* axes, we validated the hypothesis stating that the error along the *z* axis comes from the FTS.

We analyzed the obtained errors in regard to the dimensions of the sensor cells. Each cell has a size of 1 cm × 1 cm, which means that if contact points are chosen at random and the system is perfectly accurate, the average error should be 2.5 mm. Thus, the error obtained for the contact positions is 1.7 mm compared to a perfectly accurate system (we recall that we assumed an ideal *z* axis so that this error is computed only from *x* and *y* positions). At the end, this experiment proved that the positions and directions of the contacts are well estimated with the generated B-spline surface, with a precision of 3% for the directions and 1.7 mm for the positions, if we consider the FTS data as a reference. This precision is lower than the precision of calibration methods developed for non-flexible tactile sensors [23] (2 mm for the 3D positions) but was achieved with a flexible tactile sensor. This result could be improved but is fair enough for the targeted use cases, as the tactile sensor is usually touched by a human hand and thus in an area larger than a single cell.

The main finding of this paper is the spatial calibration method for flexible tactile sensors, which can be adjusted to match complex shapes, which is not in the existing literature. This method proved the accurate approximation of the actual shape of the sensor and that estimation of the contact applied to the tactile sensor in 3D is accurate enough for the targeted applications. The proposed method requires a user-defined set of planes and a 3D mesh as input. Thus, it is not as automatic as other surface approximation methods [24,29,30,31]; however, it allows the user to finely tune the calibration, even for complex shapes. Currently, the method has been validated on a smooth surface but can take advantage of its configuration capabilities to be used on more complex surfaces. We believe that this calibration method is a necessary step for the deployment of flexible tactile sensor on robots for pHRI applications, as it fully takes advantage of the flexible property of these sensors and avoids the use of dedicated sensor supports.

In addition to the calibration, a development work has been made on the integration of the tactile sensor on the humanoid robot. It includes the development of the tactile information framework to handle sensor data and its integration to the control framework mc_rtc. Further work will target the development of dedicated control tasks using the tactile sensor in a closed loop and pHRI experiments involving human-humanoid physical contact. Regarding the calibration, integration of this tactile sensor on other parts of humanoid robots may be performed if required for pHRI experiments. In addition, as the sensor we are using can be stretched [32], an improved version of our calibration method will be developed to handle stretchable tactile sensors. Other improvements of the present method can be envisioned as well to target high-resolution tactile skins (as the skins used for fingertips) or more complex surfaces, for example, with strong edges. As a conclusion, we consider that the use of a flexible tactile sensor can be useful in pHRI applications; the present and future works are a step forward in their deployment.

## 6. Conclusions

In this paper, we proposed a spatial calibration method of matching the position on flexible sensing arrays and the position on the robot, to which the sensor is attached. We used a B-spline surface to parameterize the curved surface of the robot and a constrained NLLS algorithm to identify the best matching surface parameters, given a mesh of the robot surface. A flexible sensing array was applied to the right wrist of the HRP-4C humanoid robot and was calibrated with the developed method. The spatial calibration was verified regarding a ground truth provided with two methods: equivalent force-applied measured with a force/torque sensor and contact position measured with motion capture. The performance of the position calibration had a mean error of 4.2 mm against the ground truth, i.e., 1.7 mm over the mean error of an ideal system, where the self-touch-based method for non-flexible sensors had a mean error of 2 mm. Both errors are below the half-length of a cell, which shows that the proposed method achieves results as good as methods designed for non-flexible sensors, but for flexible sensors.

## Figures and Tables

**Table 1:** Dimensions, physical properties and performance parameters of the flexible tactile sensor.

Property	Value	Comments
Dimensions	6 × 22 cells	-
Cell size	1 cm × 1 cm	-
Sensing modality	Capacitive	-
Young’s modulus	13 MPa	For strains under 10%
Sensing ranges	35–45 pF	Due to compression of the void layer in the sensor [32].
	45–125 pF	Due to compression of the elastic body [32].
Sensitivity	10 dgt./kPa	In the weak sensitivity range (35–45 pF).
Limit of detection	200 kPa	In the strong sensitivity range (45–125 pF).
Response time	100 ms	For an elastic ball hit against the sensor. See Figure S1 (2).
Recovery time	5.1 s	For a 10 kPa load applied for 2 s. See Figure S1 (3).
Hysteresis	Not detected	Not detected with the applied loads. See Figure S1 (4,5).

**Table 2:** Geometric distance and Hausdorff distance between the mesh M and the B-spline surface S, with varying parameters *n* and *m*.

Parameters		Geometric Distance (mm)			Hausdorff Distance (mm)			Generation
*n*	*m*	min	max	mean	min	max	mean	time (ms)
5	5	0.001	3.820	0.987	0.000	3.820	1.030	344
5	10	0.001	3.856	0.824	0.000	3.856	0.869	414
10	5	0.001	3.820	1.006	0.000	3.820	1.036	598
10	10	0.001	3.856	0.854	0.000	3.856	0.885	766

## Data Availability

The data presented in this study are available in Table S1.

## Supplementary Materials

The following supporting information can be downloaded at: https://www.mdpi.com/article/10.3390/s23094569/s1. Figure S1: Output curves of the flexible tactile sensor. Table S1: Positions and directions obtained during the experiment from the force/torque sensor (FTS), the motion capture system and the calibration method. Figure S2: Photographs of the elements of the experimental setup: (1) Support including the force/torque sensor, wrist and tactile skin. (2) Wand. (3) Motion capture cameras. Algorithm S1: Python code to verify the geometric computation of equivalent forces through simulation, in which the geometric expression is compared to the one obtained with the definition of equivalent force on 10,000 randomly generated force/couple systems. Figure S3: Visualization of the sorted absolute direction errors obtained with Algorithm S1.

## Abbreviations

The following abbreviations are used in this manuscript:

HRI	Human–Robot Interaction
pHRI	Physical Human–Robot Interaction
ROS	Robot Operating System
SPP	Serial Port Profile
TC	Tactile Contact
QP	Quadratic Programming
NLLS	Non-Linear Least Squares
NURBS	Non-Uniform Rational B-Spline
FTS	Force/Torque Sensor
