# Supplementary material for: Spatial Calibration of Humanoid Robot Flexible Tactile Skin for Human–Robot Interaction
Source: Sensors (Basel). 2023 May 8;23(9):4569. doi: 10.3390/s23094569 (PMC10181520; doi:10.3390/s23094569)
Supplement: Supplementary file 1 [file sensors-23-04569-s001.zip › sensors-2346017-supplementary.pdf]

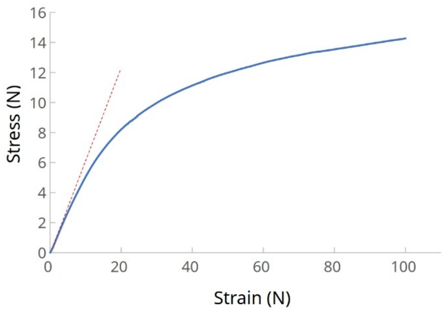

(1) S-S curve of the sensor.

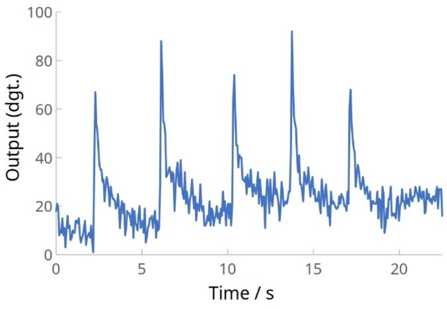

(2) Sensor output curve when an elastic ball is thrown at the sensor.

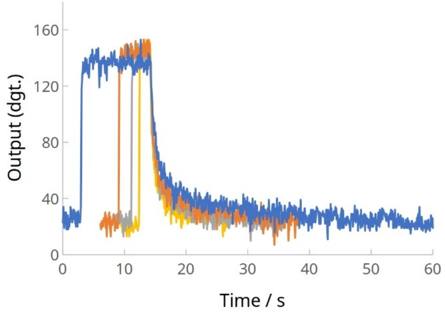

(3) Curves of sensor output when a 10 kPa load is applied to the sensor for 2, 3, 5, and 10 seconds and then removed.

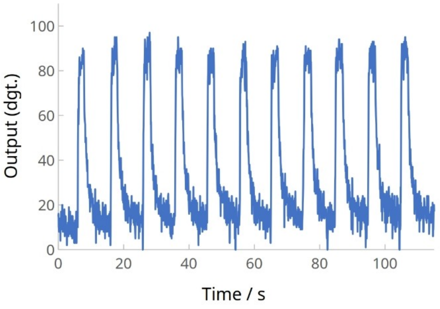

(4) Sensor output curve when a load of 10 kPa is repeatedly applied to the sensor.

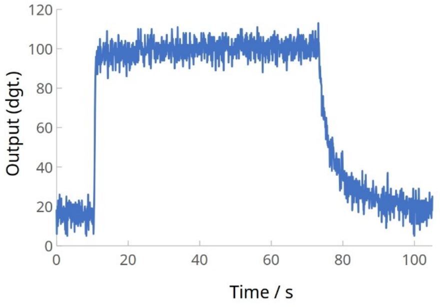

(5) Sensor output curve when a load of 10 kPa is continuously applied to the sensor for about 1 minute.

Figure S1. Output curves of the flexible tactile sensor.

Table S1. Positions and directions obtained during the experiment from the force/torque sensor (FTS), the motion capture system and the calibration method.

Touch No. i	Dimension	Contact point (m) FTS**	Motion capture	Calibration	Direction of the force (1)* FTS	Calibration
1	x	0.01971	0.0286	0.02623	0.98729	0.99143
	y	0.00413	-0.03111	-0.00334	-0.13923	-0.13061
	z	-0.0251	-0.03648	-0.01974	0.07667	-1.2e-16
2	x	-0.01548	-0.00315	2.0e-18	0.15171	0.14093
	y	-0.02642	-0.04029	-0.03398	-0.97582	-0.99002
	z	-0.0076	0.00399	-0.00252	0.15736	1.2e-17
3	x	-0.01928	-0.0298	-0.02889	-0.99337	-0.99632
	y	-0.00953	-0.02305	-0.00869	-0.06978	-0.0857
	z	0.00578	0.02227	0.01469	0.09139	4.0e-18
4	x	0.03162	0.02877	0.02748	0.89551	0.99951
	y	0.01323	-0.01978	0.01145	-0.02574	-0.03129
	z	-0.01306	-0.05245	-0.01974	0.44429	1.1e-16
5	x	0.02017	-0.01164	0.02505	0.93427	0.97668
	y	-0.00698	-0.05126	-0.01017	-0.20244	-0.21468
	z	-0.02241	-0.04435	-0.03158	0.29353	1.1e-17
6	x	-0.02731	-0.02617	-0.02736	-0.86939	-0.95007
	y	-0.01318	-0.00974	-0.01665	-0.29599	-0.31202
	z	-0.012	-0.006	-0.01974	0.39567	-2.1e-17
7	x	0.02164	-0.02584	0.01959	0.62588	0.81242
	y	-0.0279	-0.06288	-0.02452	-0.44368	-0.58308
	z	-0.02817	-0.04589	-0.01974	0.64143	-1.8e-17
8	x	0.01113	0.01648	0.01542	0.38611	0.46451
	y	0.02856	-0.01077	0.03397	0.77114	0.88557
	z	0.0071	0.01157	0.01469	0.50622	-4.2e-17
9	x	0.00761	0.02861	0.02304	0.80027	0.9479
	y	-0.01559	-0.01248	-0.01734	-0.23323	-0.31856
	z	-0.00212	0.01592	0.00608	0.55243	-1.4e-17
10	x	0.03707	0.04563	0.02505	0.96488	0.97668
	y	0.0019	0.00237	-0.01017	-0.22581	-0.21468
	z	0.02257	0.01409	0.00608	0.76573	1.1e-17
11	x	-0.02303	-0.02135	-0.02832	-0.9819	-0.991
	y	0.00752	-0.00228	0.00649	0.1604	0.13386
	z	0.00147	-0.00847	0.00608	0.54359	2.1e-16

* The directions are normalized to obtain unit vectors.

** The error on z axis has been removed to compute the intersection points from FTS data.

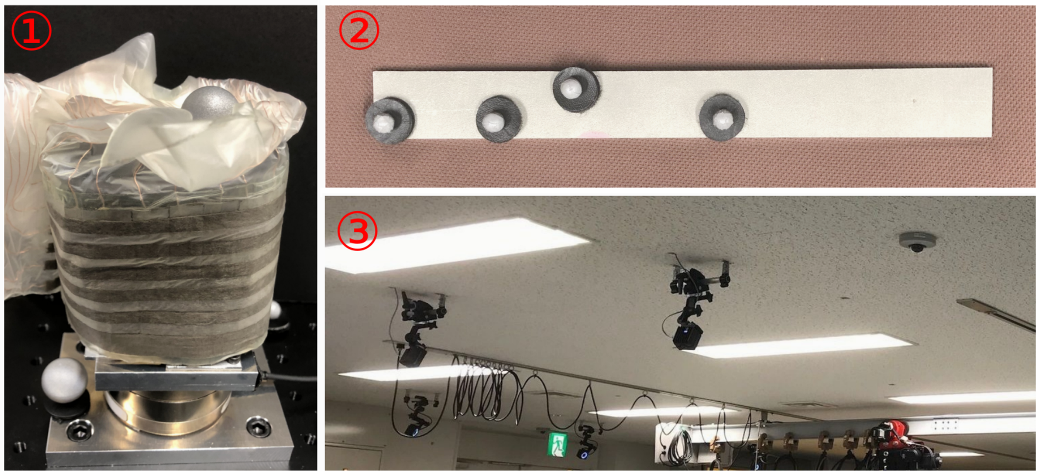

Figure S2. Photographs of the elements of the experimental setup: (1) Support including the force/torque sensor, wrist and tactile skin. (2) Wand. (3) Motion capture cameras.

Algorithm S1. Python code to verify the geometric computation of equivalent forces through simulation, in which the geometric expression is compared to the one obtained with the definition of equivalent force on 10000 randomly generated force/couple systems.

```python
import numpy as np
from scipy.optimize import minimize_scalar

# Fixing random state for reproducibility
np.random.seed(1592348)

# Array of errors
errs = np.zeros(10000, dtype=np.longdouble)

# Function to compute the application point with geometric method
def application_point_geometric(F, N):
    return (1 / np.linalg.norm(F)) * np.linalg.norm(N - ( np.dot(N, F) / np.linalg.norm(F)**2 )*F ) * ( np.cross(F, N) / np.linalg.norm(np.cross(F, N)) )

# Generate 10000 random tests
for i in range(10000):
    # Generate a random unit vector
    u = np.random.rand(3)
    u = np.array(u, dtype=np.longdouble)
    u /= np.linalg.norm(u)

    # Generate a random force vector  perpendicular to u
    resultant_force = np.random.rand(3)
    resultant_force = np.array(resultant_force, dtype=np.longdouble)
    resultant_force -= np.dot(resultant_force, u) * u
    resultant_force /= np.linalg.norm(resultant_force)

    # Generate a random couple vector perpendicular to u and resultant_force
    resultant_couple = np.random.rand(3)
    resultant_couple = np.array(resultant_couple, dtype=np.longdouble)
    resultant_couple -= np.dot(resultant_couple, u) * u
    resultant_couple -= np.dot(resultant_couple, resultant_force) * resultant_force
    resultant_couple /= np.linalg.norm(resultant_couple)

    # Origin of the system
    point_O = np.array([0, 0, 0], dtype=np.longdouble)

    # Define a function to minimize
    def distance_to_resultant_force(d):
        perpendicular_force = resultant_force + (d * (resultant_couple / np.linalg.norm(resultant_couple)))
        return np.linalg.norm(np.cross(point_O, perpendicular_force)) / np.linalg.norm(perpendicular_force)

    # Minimize the function to find the perpendicular distance d
    res = minimize_scalar(distance_to_resultant_force, tol=0.0001)

    # Compute the equivalent force system
    equivalent_force = resultant_force
    equivalent_moment = np.cross(point_O, equivalent_force) + resultant_couple + (res.x * (resultant_couple / np.linalg.norm(resultant_couple)))

    # Find the point of application of the force
    point_of_application = np.cross(equivalent_moment, equivalent_force) / np.linalg.norm(equivalent_force)**2

    # Find the point of application of the force
    point_of_application_geom = application_point_geometric(F=resultant_force, N=resultant_couple)

    # Compare vector parallelity
    v1 = point_of_application_geom
    v2 = point_of_application
    err = np.abs( np.dot(v1,v2)/(np.linalg.norm(v1)*np.linalg.norm(v2)) + 1 )

    # Add error to table
    errs[i] = err

# Print sorted errors
errs = np.sort(errs, axis=0)
print(errs)
```

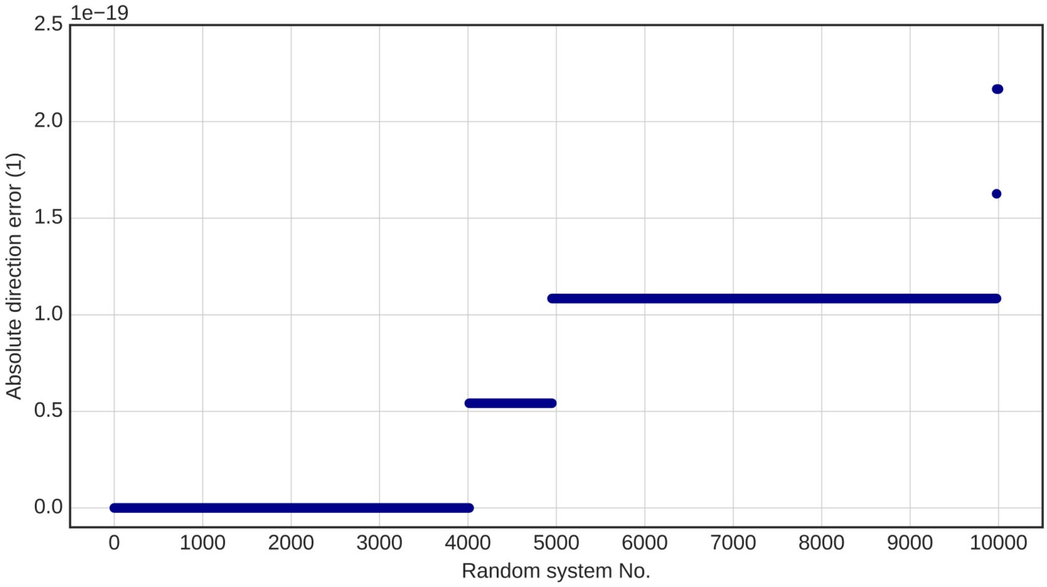

Figure S3. Visualization of the sorted absolute direction errors obtained with Algorithm S1.
